# The Total Artificial Heart: A Historical Perspective

**DOI:** 10.3390/jcm14176290

**Published:** 2025-09-05

**Authors:** Daniele Masarone, Rita Gravino, Luigi Falco, Dario Catapano, Fabio Valente, Cristiano Amarelli, Claudio Marra, Michelle Kittleson, Pierino Di Silverio, Emilio di Lorenzo

**Affiliations:** 1 Department of Cardiology, AORN dei Colli-Monaldi Hospital, 80131 Naples, Italy; 2 Department of Cardiac Surgery and Transplant, AORN dei Colli-Monaldi Hospital, 80100 Naples, Italy; 3 Department of Cardiology, Smidt Heart Institute, Cedars-Sinai Medical Center, Los Angeles, CA 90048, USA; 4 Campania Region Transplant Center, AORN dei Colli-Monaldi Hospital, 80131 Naples, Italy

**Keywords:** total artificial heart, advanced heart failure, history of medicine

## Abstract

Scientists, physicians, and engineers have long endeavored to develop a device capable of replacing the function of the heart. The objective is to create a machine or pump for patients suffering from advanced biventricular heart failure, where survival until transplantation is feasible. These total artificial hearts are designed to sustain the patient until a donor heart becomes available. This review aims to present an overview and historical context of the development of the total artificial heart, analyze the currently utilized devices, and briefly discuss future technological innovations in total artificial heart systems.

## 1. Introduction

Since ancient Greek times, the heart has been recognized as a vital organ crucial for human survival. By 500 BC, Empedocles described the heart as the most important organ due to its role in distributing “life” through the vessels [1]. Modern physiology has shown that the heart is essential for life [2]. As a result, researchers and doctors have long aimed to develop devices that can replace a failing heart. Initial efforts focused on creating temporary machines or pumps for patients with biventricular advanced heart failure, who depend on such devices until a transplant becomes available. These devices provide essential support while awaiting a donor heart. Subsequently, attention shifted toward designing permanent devices, enabling patients to live longer without needing a heart transplant [3]. This historical overview aims to trace the development of the total artificial heart (TAH), which supports patients with advanced biventricular heart failure either awaiting a transplant or ineligible for one. In addition, the novel TAHs will be reviewed, and future devices will be briefly discussed.

## 2. The Beginning

**Figure 1:**
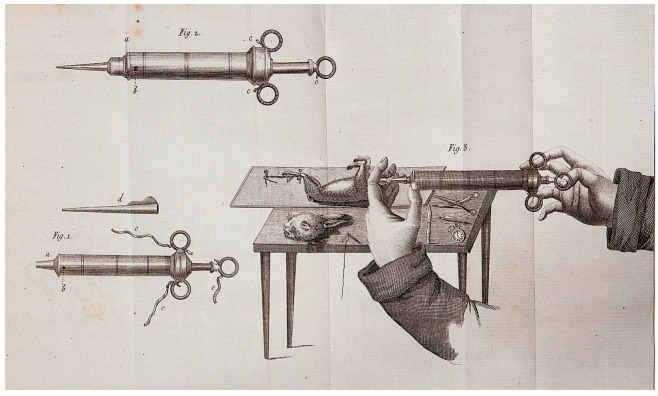
The components of the syringe that could replace the heart’s function and its use, as depicted in the book by César Julien Jean Legallois in 1812 (see ref. [4]).

The concept of extracorporeal circulation was pioneered by César Julien Jean Legallois, a French physician and physiologist, who, in 1812, published a monograph titled *Experiments on the principle of life, particularly on the movements of the heart, and on the seat of this principle* [4]. Notably, he hypothesized that the heart’s pumping function could be substituted by a syringe that is continuously supplied with natural or artificially produced arterial blood, aiming to sustain cerebral and end-organ perfusion (Figure 1).

Although this hypothesis may appear futuristic, it was not considered due to its divergence from established principles of human circulatory physiology. These principles are based on the existence of multiple chambers designed to accommodate arterial and venous blood, as well as valves functioning unidirectionally to regulate blood flow to the body.

**Figure 2:**
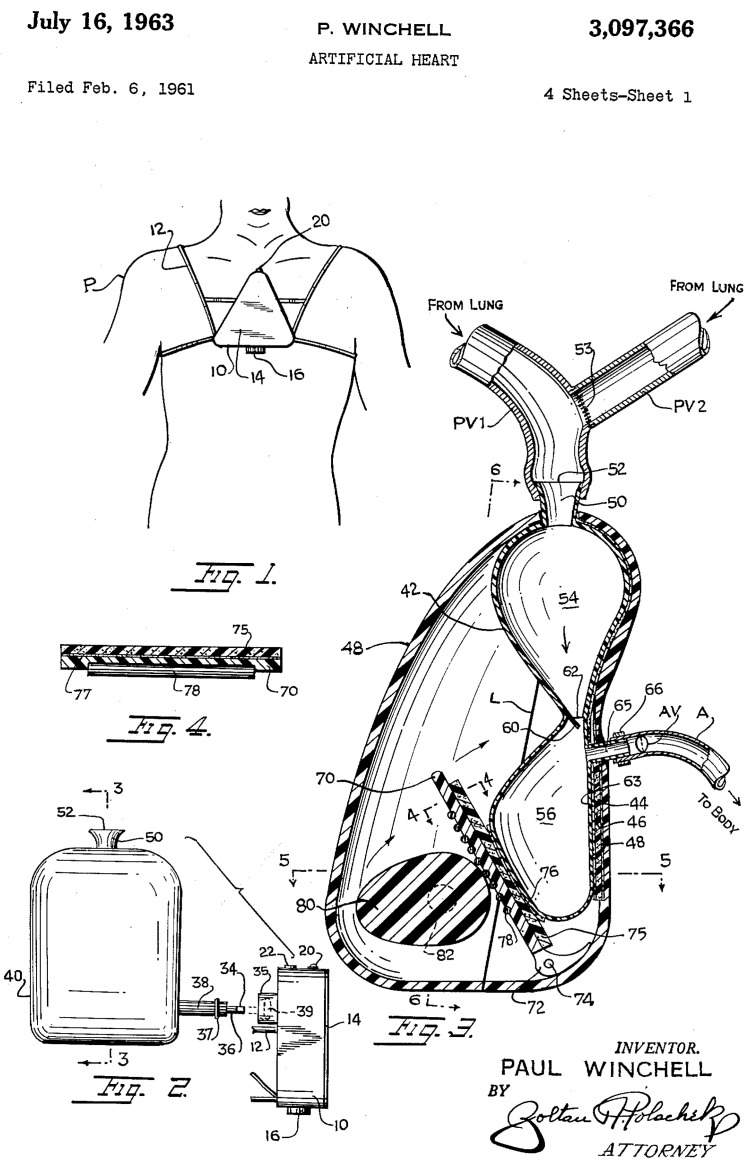
The Winchell TAH.

Subsequently, in 1937, Vladimir Demikhov, a Russian scientist and pioneer in organ transplantation, implanted the first biventricular support device in a canine subject, successfully maintaining its life for 5.5 h [5]. In 1949, the development of a precursor to the modern artificial heart pump was developed by physicians William Sewell and William Glenn. The pump, known as the Sewell Heart Pump, was constructed from an Erector© Set, glass cylinders, a cannula, and rubber tubing. The first implantation of this pump took place in June 1949 in a dog with advanced heart failure; the procedure was successful, and the dog remained alive for one hour [6]. On 12 December 1957, Willem Johan Kolff, widely recognized as a pioneering figure in the field of artificial organs, and Dr. Tetsuzo Akutsu from the Cleveland Clinic in the United States successfully implanted the first TAH into a dog, which survived for 90 min [7]. In 1958, Domingo Liotta commenced studies on heart replacement using a TAH in Lyon, France, and continued in 1959–1960 at the National University of Córdoba, Argentina. He presented his work at the meeting of the American Society for Artificial Internal Organs held in Atlantic City in March 1961. During this presentation, Liotta described the implantation of three types of orthotopic TAHs (located within the pericardial sac) in dogs, each utilizing different external energy sources: an implantable electric motor, an implantable rotary pump with an external electric motor, and a pneumatic drive pump [8]. Paul Winchell designed a TAH model with help from Henry Heimlich, the inventor of the Heimlich maneuver; this prototype used a cam-driven roller mechanism to compress flexible bags containing blood (Figure 2).

**Figure 3:**
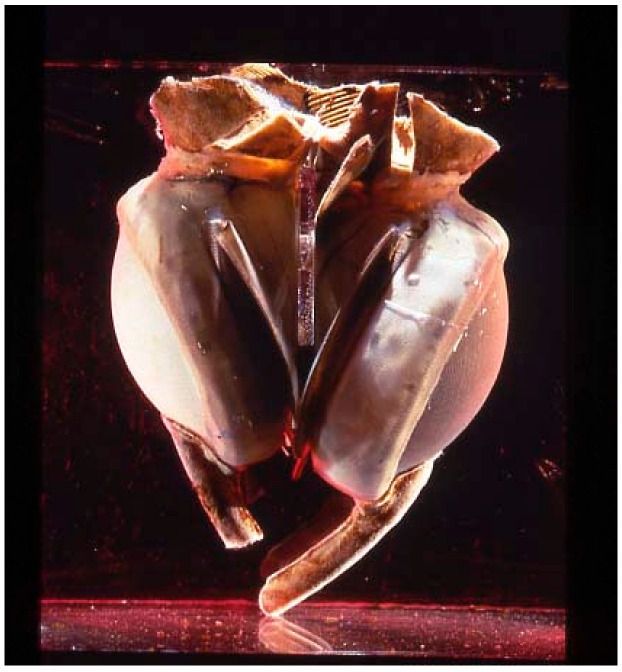
The Liotta–Cooley TAH.

**Figure 4:**
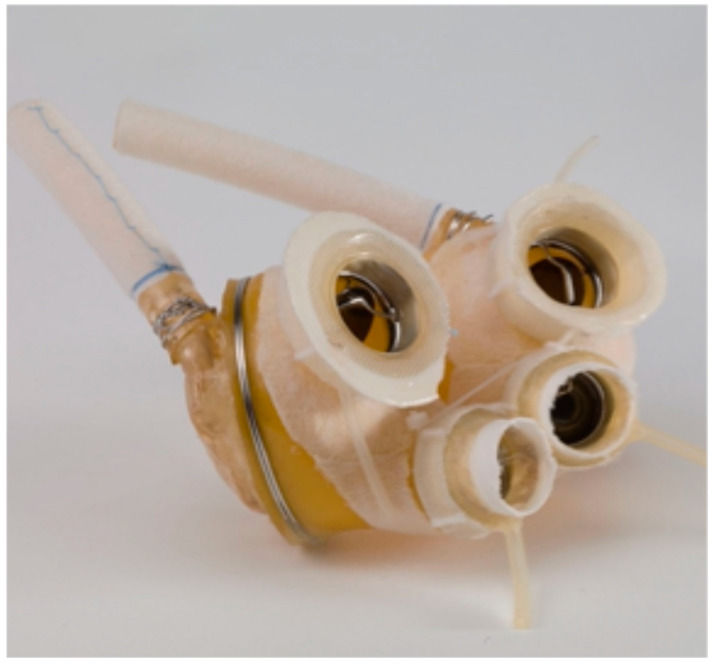
The Akutsu III TAH.

On 4 April 1969, Domingo Liotta and Denton A. Cooley replaced a dying man’s heart with a TAH at the Texas Heart Institute in Houston [9,10]. This TAH, designed by Liotta, consisted of two external pneumatic pumps like models in use today (Figure 3). The patient was supported mechanically for about 64 h before transplantation. In 1981, Cooley performed the second implantation of a TAH (the Akutsu III; Figure 4) in a 36-year-old man with post-cardiac surgery shock. The patient underwent transplantation after 55 h of support.

Dr. Robert Jarvik is renowned for developing the first successful permanent TAH. In 1982, surgeons at the University of Utah implanted the first permanent artificial heart into a 61-year-old patient named Barney Clark. Jarvik had been working there since 1971 in the artificial organ development division, collaborating with Dr. Willem Kolff. As Dr. Kolff often named artificial hearts using the names of researchers involved, and since this was the seventh prototype by the team, the TAH was called the Jarvik 7 [11]. Barney Clark lived for 112 days and died on 23 March 1983, from multiple organ failure. DeVries then moved his practice to Humana Hospital Audubon in Louisville, Kentucky, to continue studying the Jarvik-7. The second patient to receive the device was Bill Schroeder on 25 November 1984. In the immediate post-operative period, his condition initially improved markedly, leading to a phone call from President Reagan [12]. Nonetheless, 19 days after the operation, he experienced the first of four strokes, and passed away on 6 August 1986, after 620 days with the TAH. Three other patients received the Jarvik-7 as a destination therapy. Murray Haydon, DeVries’ third patient, received a Jarvik-7 on 17 February 1985. Haydon died from infection and kidney failure on 19 June 1986, after 488 days. On 7 April 1985, Dr. Bjarne Semb from Stockholm’s Karolinska Hospital implanted a Jarvik-7 in Swedish businessman Leif Stenberg. Stenberg lived 229 days without major problems but died from a stroke on 21 November 1985 [13]. Jack Burcham was DeVries’ fourth and last patient to receive a Jarvik-7 as a destination therapy. He received the device on 14 April 1985, but died on 25 April 1985, due to complications related to bleeding, and kidney failure.

Although the initial Jarvik 7 implants should be regarded solely as surgical successes rather than clinical successes, owing to the poor outcomes observed in patients undergoing the procedure, these interventions were crucial for the subsequent development of the Jarvik 7. Although they may be viewed as morally questionable as “human experiments,” they were, in fact, conducted on patients who, according to the knowledge available at the time, had no alternative therapeutic options. Therefore, at the time TAH implantation represented a hope for survival for another few months or until heart transplantation, albeit often with a suboptimal quality of life, as exemplified in 1981 by the patient in whom Dr. Cooley implanted the Akutsu III TAH.

The Jarvik 7 was equipped with two pneumatic air pumps that simulated cardiac function at a rate ranging from 40 to 120 beats per minute [14]. Each chamber incorporated a polyurethane disc mechanism responsible for propelling blood from the inflow valve through the device [14]. To connect the TAH to the patient’s natural atria, cuffs were employed, linked via reinforced polyurethane transmission lines coated to facilitate tissue integration. These lines were inserted through the patient’s left side. An electronic power unit, comparable in size to a refrigerator, supplied electricity to enable the device’s operation. This unit also regulated critical functions such as pumping frequency and pressure through a combination of electricity, compressed air, and vacuum systems. The device was later renamed the Cardiowest Total Artificial Heart. This change occurred after Symbion, the original manufacturer, ceased production in 1990 due to non-compliance with FDA standards. MedForte Research acquired the rights from Symbion and collaborated with the University Medical Center in Tucson, Arizona, to develop the CardioWest Heart. Consequently, the device was renamed the Cardiowest TAH in 1991 [15].

The AbioCor TAH is recognized as the first “autonomous internal artificial heart.” Researchers dedicated 30 years to studying and testing this device, including from its initial human use in 2001 to FDA approval in 2006 [16]. Its distinctive feature was that it required no subcutaneous connections, meaning patients were not connected to “external machines for the TAH to function.” Weighing 2 pounds, the device included four implanted components: an electronic controller, a chest unit, a lithium battery, and a transcutaneous energy transmission device, which also contained “two artificial ventricles and the valves” [17]. The system used a hydraulic pump driven by a motor to mimic a human heartbeat. The internal battery was recharged by the TET and an external battery, with the transcutaneous energy transmission transmitting energy through the skin [18]. The internal battery lasted up to 30 min, while the external battery could operate for around 4 h. Only one patient received the AbioCor as a bridge to transplantation, but it was later withdrawn from the market due to complications including thromboembolism, particularly stroke related to the presence of a thrombus on the atrial struts and suction events due to an incorrect balance between the two ventricles [19].

## 3. The Modern Era

Bioengineers and researchers have progressively addressed the primary limitations of early TAH models, specifically device size, hemocompatibility, and infections, leading to the advancements observed in current models. Technological advancements have enabled the production of smaller components that perform equivalent functions, resulting in a substantial reduction in device size, particularly concerning external controllers. Hemocompatibility has been markedly improved through the application of technologies like those employed for bioprosthetic heart valves, namely the utilization of xenogenic pericardial tissue treated with glutaraldehyde. In contemporary TAHs, the blood-contacting layer is coated with this tissue, significantly enhancing hemocompatibility [20]. Furthermore, in 1988, four research groups initiated the development of permanent, transcutaneously powered, fully implantable electromechanical TAHs with recharging capabilities based on transcutaneous energy transfer technology, aiming to minimize infections associated with percutaneous drivelines. Nevertheless, despite the availability of this technology for some time, no TAHs currently incorporate this system, as they remain dependent on a percutaneous driveline for battery power.

### 3.1. Syncardia Temporary TAH

**Figure 5:**
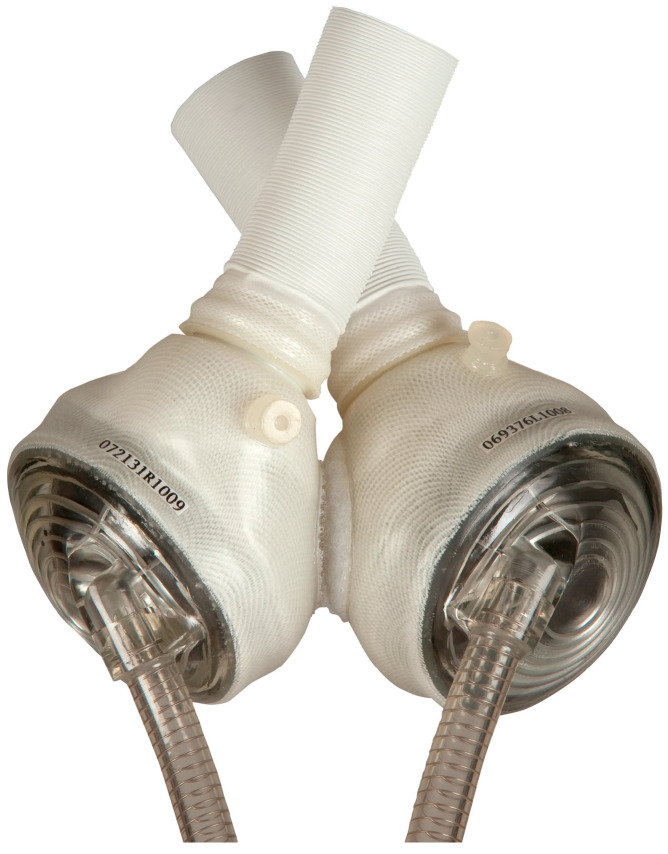
The Syncardia temporary TAH.

In 2010, the CardioWest was rebranded as the SynCardia Temporary TAH. The technology of this TAH closely resembles that of the Akutsu III and Jarvik 7 models. SynCardia is a pneumatically operated TAH comprising two independent ventricles that simultaneously collect and expel blood in a pulsatile manner (Figure 5). A four-layer polyurethane membrane separates the blood within the ventricle from the pneumatic chamber. The pressures within the pneumatic chamber are generated by an external pump connected via percutaneous transmission lines. SynCardia is the first TAH to obtain CE approval, in 1999, and FDA approval, in 2004, for temporary use as a bridge to heart transplantation in eligible patients at imminent risk of death due to end-stage biventricular failure. A pivotal clinical trial demonstrated survival rates of 79% compared to 46% in control groups and one-year and five-year post-transplantation survival rates were 86% and 64%, respectively [21]. To date, the Syncardia temporary TAH has been implanted in over 2.100 patients worldwide, and a recent meta-analysis including four studies and 299 patients showed an overall survival of 85.8% at 1 month, 76.3% at 3 months, 63% at 6 months, and 42.2% at 12 months [22]. A more recent single-center experience including 196 patients who underwent a Syncardia TAH implant showed that survival rates at 1, 6, and 12 months were 72%, 41%, and 34%, respectively [23].

Regarding safety, the primary complications after Syncardia implantation include the following:

- Infections: These are the most common adverse events following SynCardia TAH implantation, with an infection rate estimated at 20–50% for systemic infections and 27% for transmission line infections [24].
- Device malfunction: This is a significant concern, with reported rates of 20.1% [25].
- Bleeding: Hemorrhagic events are quite common, occurring in about 15% of cases [24].
- Stroke: Cerebrovascular accidents happen in roughly 10% of cases [26].

### 3.2. The CARMAT Aeson

**Figure 6:**
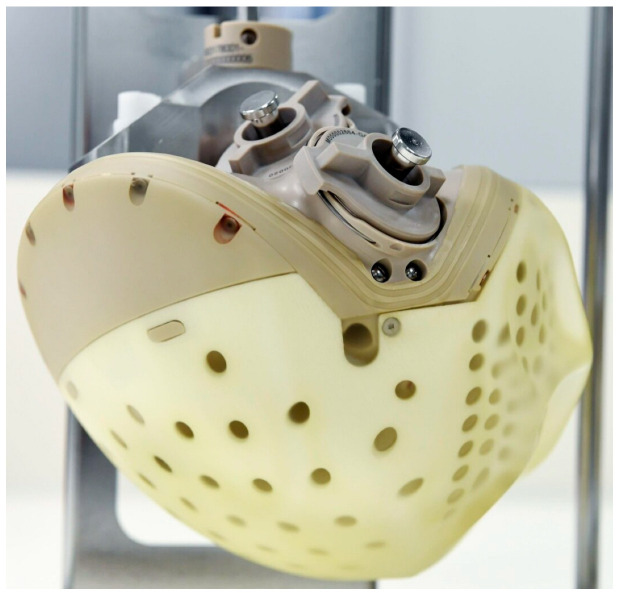
The CARMAT Aeson TAH.

The CARMAT Aeson is a pioneering total artificial heart (TAH) developed entirely from biocompatible materials which received approval from European regulatory authorities in 2020 for patients with advanced heart failure as a bridge to transplant [27]. The TAH features a pneumatic, pulsatile system equipped with four biological valves (Figure 6). This device comprises two chambers, each representing a ventricle with a maximum volume of 60 mL [28]. Unidirectional blood flow is maintained by four bioprostheses: two located at the atrial suture rings and two separating the ventricles from the Dacron vascular prostheses, which are subsequently anastomosed to the aorta and pulmonary artery. The device connects to a portable control system via an 8 mm diameter transmission cable. This external system provides mobility and autonomy, weighing approximately 4 kg, and includes a controller and two battery compartments, each capable of sustaining approximately four hours of operation [29]. The device generates a pulsatile blood flow designed to mitigate shear stress, damage to von Willebrand factor multimers, and platelet activation, and thereby reduce gastrointestinal bleeding and thromboembolic events [30]. Embedded pressure and volume sensors enable the device to self-regulate cardiac output, which can range from 2 to 9 L/min, in accordance with the patient’s physiological demands. The CARMAT Aeson also has internal sensors that facilitate auto-adaptation to preload and afterload alterations by modifying the frequency (35–150 bpm) and systolic volume (55–60 mL). The ventricular pressure sensor assesses intracardiac pressure, providing real-time data on venous return. The system’s self-adjusting mechanism responds to the Frank–Starling law: elevated pressure prompts an increase in the pacemaker frequency, while low afterload results in an increased systolic ejection velocity. Variations in filling time, ejection time, and end-diastolic volume correspondingly adjust cardiac output. The stroke volumes of each ventricle are continuously adjusted to prevent imbalance between the mean inflow pressures of the left and right sides [31]. When this difference exceeds 2.5 mmHg, the stroke volume of the right ventricle is automatically reduced to decrease the left inflow pressure. Conversely, if the difference is less than 2.5 mmHg, the right systolic volume is maximized. This regulation aims to produce a cardiac output responsive to changes in venous return [32]. According to a company announcement, more than 100 implants were performed in patients with advanced heart failure [33].

## 4. The Future

### 4.1. Realheart TAH

The Realheart TAH is a four-chamber total artificial heart currently in development. It showed improved survival rates in animal testing in 2022, increasing from one to four days, along with better hemodynamic status. After implantation, the animals could stand and eat food [34]. Furthermore, the Realheart TAH met key performance criteria, including no hemolysis, no thromboembolic events, high pumping capacity, and good right–left balance [35]. In January 2025, the Food and Drug Administration granted humanitarian use device status to the Realheart TAH, opening the way for its use in treating patients with advanced biventricular heart failure who have limited options and need TAH support [36].

### 4.2. Cleveland Clinic Continuous Flow TAH

The Cleveland Clinic Continuous Flow TAH is a pulsatile, valveless device with a single moving component and can supply systemic and pulmonary perfusion from one implanted unit. It consists of two impellers driven by a motor. The rotor, with impellers on opposite ends, is supported radially by a hydrodynamic bearing. The outlet pathways—systemic and pulmonary circulation—are self-regulated through passive axial movement of the rotor in response to atrial pressure differences [37]. At present, the development of this TAH is still in the technology implementation phase, based on its use in animal models.

### 4.3. BIVacor TAH

BiVacor constitutes a titanium-constructed biventricular rotary blood pump designed as a short- and long-term TAH. BiVacor integrates a rotary blood pump with magnetic levitation technology to generate a distinctive contactless rotating disc that effectively pumps blood into the systemic and pulmonary circulation. Rapid cyclic variations in pump speed induce pulsatile flow, while the ample spacing between the blood and device components minimizes shear stress on the blood. Over the past decade, the development of the BiVacor system has resulted in multiple versions of the device, which have been implanted in over 30 chronic animal studies. In 2023, the FDA approved the device. In March 2025, the BiVacor TAH was successfully implanted in the first patient in Australia; the patient remained on hemodynamic support with the TAH for 100 days before receiving a heart transplant. Subsequently, four additional patients received BiVacor implants during a preliminary FDA study to assess the safety and feasibility of BIVACOR as a bridge to transplant for patients with severe biventricular dysfunction. In May 2025, BiVacor received Breakthrough Device Designation from the Food and Drug Administration, facilitating a faster pathway for the application of this TAH in patients with advanced biventricular heart failure.

### 4.4. LIMO TAH

In the last five years researchers have developed a new compact TAH prototype called the LIMO (Less In, More Out) TAH, which uses an efficient soft fluidic transmission system. This technology works by reducing the actuator’s volume and improving energy transfer, resulting in a more compact device design and increased efficiency. The initial prototype, made with thin-walled bag actuators, has shown promising results in vitro, achieving a cardiac output of 5.9 L per minute [38]. Based on these findings, researchers are now exploring the possibility of testing the LIMO TAH in living models.

### 4.5. Hybrid Heart TAH

The hybrid heart TAH represents the latest prototype of a soft robotic hybrid TAH, wherein the propulsion mechanism is derived from soft robotics, and the interior lining consists of the patient’s own cells. The device incorporates a pneumatic actuator (septum) situated between the two ventricles and is coated with supramolecular polymeric materials designed to enhance antithrombotic and tissue engineering properties. In vitro analyses demonstrate that the hybrid heart can pump 5.7 L per minute, replicating the adaptive functions of the native heart. Proof-of-concept studies conducted in rats and an acute goat model have confirmed the potential of the hybrid heart for clinical application and its superior biocompatibility [39].

## 5. Unmet Needs of TAHs

The main limitation of current TAHs is their dependence on an external power supply for the pump, which makes them vulnerable to infections that can spread systemically. As a result, researchers are concentrating on creating fully implantable and autonomous TAHs. Nonetheless, several challenges need to be overcome, including ensuring sufficient durability, reducing risks of thromboembolism and hemolysis, maintaining the balance between pulmonary and systemic circulation, and adapting to limitations in women, small adolescents, and children.

## 6. The Left Ventricular Assist Device: The Little Brother of the TAH

Unlike TAHs, left ventricular assist devices (LVADs) can support the left ventricle’s function, but not the failing heart’s biventricular function [40]. Although they are made of similar materials and have an external controller to regulate blood flow, they work slightly differently. The LVAD uses a battery-powered pump to continuously pull blood from the left ventricle and push it into the aorta, where it circulates throughout the body. This makes the LVAD smaller and less prone to side effects. Thanks to modern technology, the only LVAD on the market—the HeartMate 3—is a fully magnetically levitated device designed for optimized hemocompatibility [41]. This greatly reduces the risk of thrombosis, with the main limitations being the need for external power and a transmission line, which can lead to superficial and deep infections. However, quality of life improves significantly with an LVAD, and the risk of adverse events decreases. As a result, LVADs have been used more often than TAHs over the past decade, despite TAHs being developed earlier.

## 7. Conclusions

During the 21st century, scientists, doctors, and engineers have made significant progress in developing TAHs. Starting with large prototypes connected to external consoles that kept patients in hospitals, advances in technology have led to fully implantable TAHs that enable patients to live outside the hospital. With current technology, a TAH’s purpose is not to replace the heart entirely but to support patients who cannot survive without it. Despite these advancements, no TAH has yet been created without defects or post-implantation issues. Nonetheless, considering the progress over the past century, we might see a TAH within the next ten years that can serve as a bridge to transplant and potentially replace cardiac function indefinitely.

## Data Availability

No new data were created or analyzed in this study. Data sharing is not applicable to this article.
